# Radiological Union and Functional Recovery Following JUST UNIC™ Fixation for Comminuted Anatomical Neck Proximal Humerus Fractures: A Single-Centre Retrospective Case Series

**DOI:** 10.7759/cureus.102258

**Published:** 2026-01-25

**Authors:** Senthilvelan Rajagopalan, Senthil Narayanan, Bijay K Sahu, Robin G Alex, Girinivasan Chellamuthu, A Ravikumar

**Affiliations:** 1 Shoulder and Elbow, MIOT International, Chennai, IND; 2 Arthroscopy, Orthopaedic Research Group, Coimbatore, IND

**Keywords:** anatomical neck, avn (avascular necrosis), just unic, partial greater tuberosity resorption, proximal humerus comminuted fractures

## Abstract

Background

Comminuted proximal humeral fractures involving the anatomical neck pose challenges in fixation stability and vascular preservation. The JUST UNIC™ (Evolutis, Briennon, France) bilboquet-inspired fixation system has been developed to address these limitations by providing metaphyseal stability with minimal periosteal disruption. This study assesses the clinical, functional, and radiological outcomes following JUST UNIC fixation in this fracture subset.

Methods

This retrospective single-centre case series included 25 patients with comminuted proximal humerus fractures involving the anatomical neck who underwent fixation with the JUST UNIC™ system between January 2020 and December 2023 at a tertiary care hospital. Clinical outcomes were assessed using the visual analogue scale (VAS), Oxford Shoulder Score (OSS), and serial range-of-motion (ROM) measurements recorded at baseline (preoperative), six weeks, three months, six months, and 12 months (final follow-up). Radiological union, greater tuberosity (GT) resorption, and postoperative complications were evaluated through serial follow-up imaging and clinical review.

Results

All fractures achieved radiological union by six months. VAS scores reduced from 8.00 ± 0.82 preoperatively to 2.00 ± 0.61 at final follow-up, while OSS increased from 4.48 ± 1.21 to 35.48 ± 1.18. Forward flexion improved from 49.12 ± 5.83° to 141.65 ± 11.99°, abduction from 49.76 ± 4.74° to 136.41 ± 12.64°, external rotation from 30.60 ± 5.86° to 65.47 ± 5.73°, and internal rotation from 29.96 ± 4.59° to 61.76 ± 6.31. GT resorption was observed in 15 patients (60%); however, statistical analysis did not detect a significant association with final VAS, OSS, or ROM outcomes. Postoperative complications occurred in four patients (16%), including one case of avascular necrosis, one varus malalignment, one partial staple prominence, and one superficial haematoma.

Conclusion

JUST UNIC fixation demonstrated reliable union, progressive functional recovery, and a low complication burden in comminuted anatomical-neck proximal humerus fractures. The implant appears effective in maintaining alignment and facilitating early rehabilitation. Larger controlled studies with long-term follow-up are warranted for comparative validation.

## Introduction

Proximal humerus fractures (PHF) are among the most frequent fractures of the upper extremity, constituting approximately 5-6% of all adult fractures and ranking among the top non-vertebral fractures in older adults [1,2]. Their incidence is rising globally as ageing populations grow and osteoporotic fractures become more common. While elderly patients often sustain PHF after low-energy falls on an outstretched hand, middle-aged individuals more commonly sustain these injuries via high-energy mechanisms such as road-traffic accidents or direct trauma [3,4].

Fracture classification and displacement status determine management. Non-displaced or minimally displaced fractures generally respond to conservative measures, whereas displaced, comminuted, or multi-part fractures often require surgical intervention [5,6]. Among surgical options, locking plates, intramedullary nails, percutaneous pinning, or arthroplasty, the standard approach for complex PHF has been open reduction and internal fixation (ORIF) using angular-stable plates. However, fractures involving the anatomical neck present a unique challenge. Fragment instability, small articular segments, and disruption of vascular supply significantly increase the risk of humeral-head ischemia and avascular necrosis (AVN). Indeed, AVN rates after fixation have ranged from 3 to 35% in published series [7,8]. Conventional locking plate fixation in anatomical neck fractures is associated with specific limitations, including difficulty in securing small articular fragments, risk of screw cut-out, varus collapse, and disruption of humeral head vascularity, all of which contribute to higher rates of fixation failure and AVN. These risks are particularly pronounced in fractures with metaphyseal comminution and medial hinge disruption. A fixation strategy that provides central load-bearing support while minimizing periosteal and vascular disruption may therefore offer biomechanical and biological advantages in this fracture subset.

In this context, a bilboquet-style fixation system “JUST UNIC™” (Evolutis, Briennon, France) offers a promising fixation option. This device consists of a humeral stem linked via a Morse taper to a humeral-head staple component. The construct is designed to capture small head fragments securely while minimizing soft tissue disruption and preserving residual vascular attachments, thus combining mechanical stability with vascular preservation. Therefore, this study aimed to (1) evaluate radiological union and greater tuberosity (GT) behaviour, (2) characterise clinical and functional recovery using VAS, Oxford Shoulder Score (OSS), and ROM, and (3) describe postoperative complications and determine whether GT resorption is associated with inferior functional outcomes following JUST UNIC™ fixation in comminuted anatomical-neck PHFs.

## Materials and methods

Study design and ethical approval

This retrospective observational case series was conducted at a tertiary care centre. Institutional Review Board approval was obtained prior to data collection (approval no.: IEC/2025/02), and all procedures adhered to the principles of the Declaration of Helsinki. Owing to the retrospective nature of the study, the requirement for individual patient consent was waived.

All patients treated between January 2020 and December 2023 for comminuted PHFs involving the anatomical neck were consecutively screened for eligibility. Inclusion criteria comprised adults aged 18-60 years with closed anatomical neck fractures associated with metaphyseal comminution, corresponding AO/OTA type 11-C2 injuries, who underwent primary fixation using the JUST UNIC™ device and had complete baseline data with a final clinical and radiological follow-up at 12 months. Fracture morphology and anatomical neck involvement were confirmed using preoperative radiographs and computed tomography.

Patients older than 60 years (managed with reverse shoulder arthroplasty according to institutional protocol), as well as those with open, pathological, fracture-dislocation, or head-splitting fractures, prior ipsilateral shoulder surgery or infection, associated neurovascular injury, or incomplete follow-up were excluded. Ultimately, 25 patients fulfilled all eligibility criteria and constituted the final study cohort, representing the complete convenience sample during the study period.

Surgical technique

**Figure 1:**
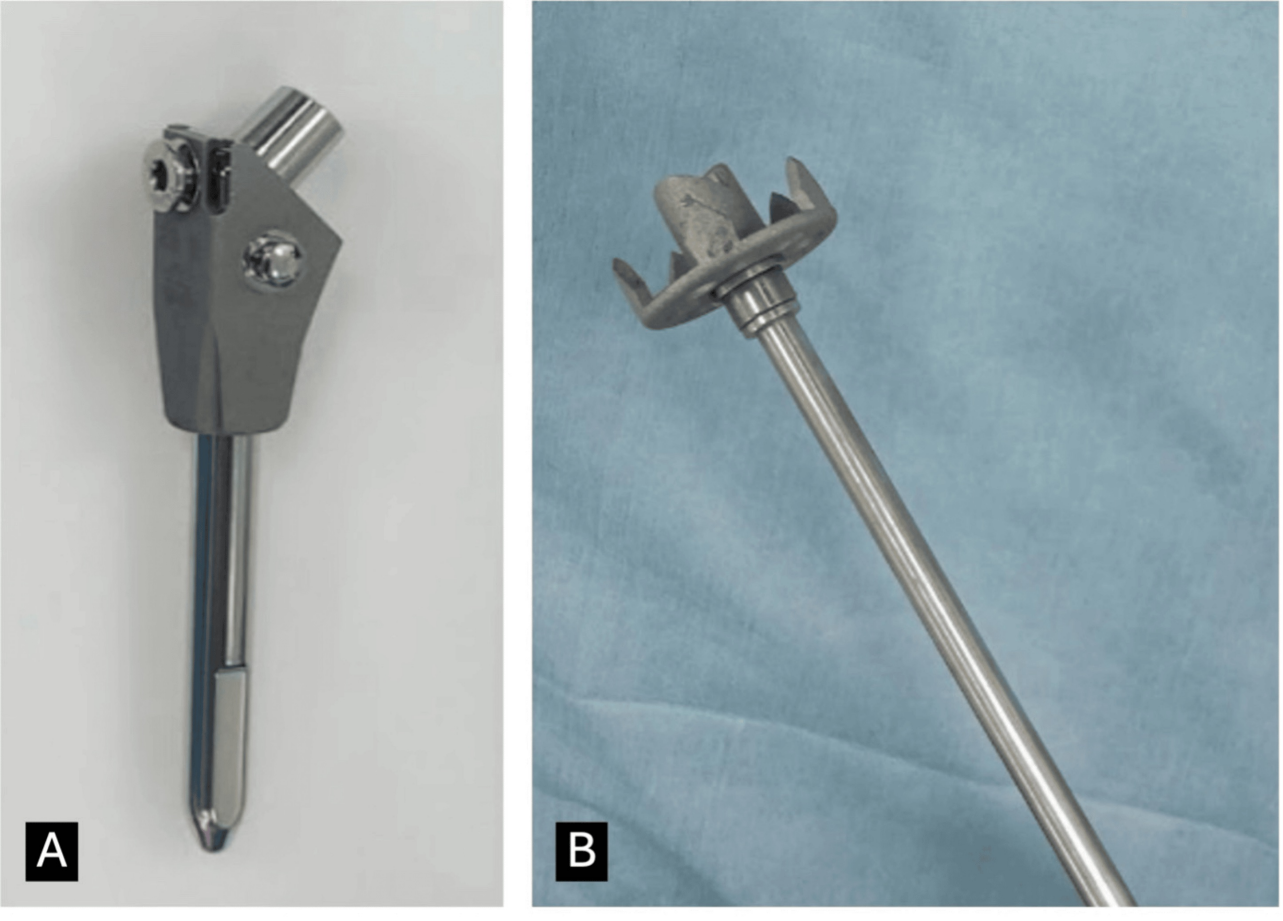
Components of JUST UNIC. (A) Humeral stem. (B) Staple for humeral head.

The JUST UNIC device is based on the bilboquet concept introduced by Doursounian et al. [9]. It consists of a staple device fitted to the humeral head, along with a humeral intramedullary stem (Figure 1). The stem and staple were connected at an angle using a Morse taper. It has the option of increasing the valgus angle intraoperatively. This allows for accurate reduction and maintenance of the normal centrum column diaphyseal angle of the humerus.

The deltopectoral approach was used in all the patients. After exposure, the greater and lesser tuberosities were secured using FiberWire sutures (Arthrex, Naples, FL) through the enthesis of the rotator cuff tendons. The humeral head was carefully exposed until its full circumference of the humeral head was visible. After an initial trial of staples of different sizes, the staple that provided maximal coverage of the humeral head was selected and impacted in position under image intensifier guidance. Subsequently, the proximal aspect of the humeral shaft is exposed and prepared. After the trial, an appropriately sized stem was implanted. The neck of the stem was engaged with the staple. The height was adjusted using a check-up forceps device to alter the valgus angle. Controlled distraction was performed under C-arm guidance until the medial arch was restored. The final position of the humeral sleeve was locked using a locking screw. The tuberosities were sutured in the anatomical position over the implant using FiberWire.

Postoperative rehabilitation

All patients were immobilized in a sling for six weeks. Early passive pendulum exercises were initiated on postoperative day 1. Active-assisted forward elevation up to 45° was allowed at four weeks and progressed to 90° by six weeks, followed by a structured program advancing to full active motion after eight to 10 weeks. Strengthening was begun after radiological evidence of union.

Clinical and functional assessments were performed at six weeks, three months, six months, and 12 months postoperatively (final follow-up), using validated outcome measures. Pain intensity was quantified using the visual analogue scale (VAS) (0-10) [10], functional status was evaluated with the Oxford Shoulder Score (OSS) [11], and shoulder mobility was assessed by measuring forward flexion, abduction, external rotation, and internal rotation with a standard goniometer. These evaluations provided a comprehensive appraisal of recovery trajectories across the first postoperative year.

Radiological evaluation included standardized anteroposterior and axillary views obtained at each follow-up. The head-shaft angle was measured on immediate postoperative AP radiographs to assess restoration of alignment. Fracture union was defined radiographically as the presence of bridging trabeculae across at least three cortices on orthogonal views. GT resorption was diagnosed by loss of cortical definition or trabecular thinning on serial images. GT resorption was assessed by comparison with immediate postoperative radiographs and defined using a pragmatic radiographic threshold, recorded when a greater than 50% reduction in the apparent vertical height of the GT fragment was observed during follow-up. This cut-off was selected to improve standardization and interobserver consistency in this retrospective analysis and was not intended as a biologically validated threshold. All cases represented partial GT resorption; no complete resorption was identified. All radiographs were independently reviewed by two fellowship-trained shoulder surgeons at two separate time points, with discrepancies resolved through consensus; intraobserver and interobserver reliability were quantified using intraclass correlation coefficients. AVN was identified based on sclerotic changes, subchondral collapse, or articular surface irregularity, while varus malalignment was defined by a neck-shaft angle <125°.

Statistical analysis

Statistical analysis was conducted using the jamovi (Jonathon Love, Damian Dropmann, and Ravi Selker, Sydney, Australia) open-access platform [12]. Continuous variables were summarised as mean ± standard deviation, and temporal changes in VAS, OSS, and ROM [13] were assessed using repeated-measures ANOVA for normally distributed data or the Friedman test for non-parametric distributions. Group comparisons between patients with and without GT resorption were performed using the Mann-Whitney U test, while categorical variables were analysed using Fisher’s exact test. A two-tailed p-value < 0.05 was considered statistically significant.

## Results

**Figure 2:**
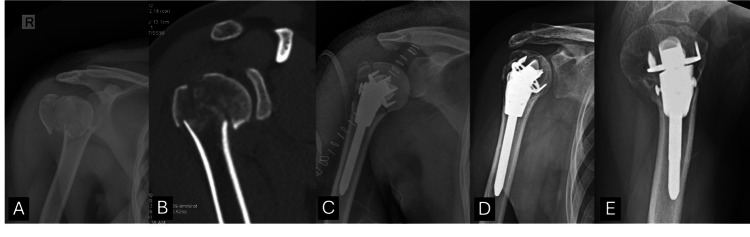
(A) Preoperative X-ray. (B) Preoperative CT image. (C) Immediate postoperative X-ray AP view. (D, E) Final follow-up X-rays AP and lateral views at 12 months showing united fracture.

A total of 25 patients were included in the study. The mean age was 45.72 ± 7.40 years, with a male predominance (20, 80%). Road-traffic accident was the most common mode of injury (19, 76%), followed by domestic falls (six, 24%). The mean duration from injury to surgery was 16.04 ± 7.83 days. The immediate postoperative head-shaft angle was 137.16 ± 8.92°, and GT resorption was present in 15 patients (60%). Preoperative VAS and OSS scores averaged 8.00 ± 0.82 and 4.48 ± 1.23, respectively, with markedly restricted ROM across all planes (Table 1). Representative imaging of fracture morphology, operative fixation, and radiological progression to union is shown in Figure 2.

**Table 1:** Patient characteristics and key radiological and clinical parameters (n = 25).

Variable (units)	Value
Age, years (mean ± SD)	45.72 ± 7.40
Gender: M/F (n, %)	Males: 20 (80%); females: 5 (20%)
Mode of injury (MOI) - n (%)	RTA: 19 (76%); fall: 6 (24%)
Time to procedure, days (mean ± SD)	16.04 ± 7.83
Immediate postoperative head-shaft angle, degrees (mean ± SD)	137.16 ± 8.92
Greater tuberosity resorption at final follow-up - n (%)	Yes: 15 (60%); no: 10 (40%)
Preoperative parameters	
VAS (0-10) (mean ± SD)	8.00 ± 0.82
OSS (0-48) (mean ± SD)	4.48 ± 1.23
Forward flexion, degrees (mean ± SD)	49.12 ± 5.83
Abduction, degrees (mean ± SD)	49.76 ± 4.74
External rotation, degrees (mean ± SD)	30.60 ± 5.86
Internal rotation, degrees (mean ± SD)	29.96 ± 4.59
Comorbidity	
HTN (n, %)	14 (56%)
DM (n, %)	10 (40%)
COPD (n, %)	2 (8%)
CKD (n, %)	1 (4%)
Dyslipidaemia (n, %)	1 (4%)

**Figure 3:**
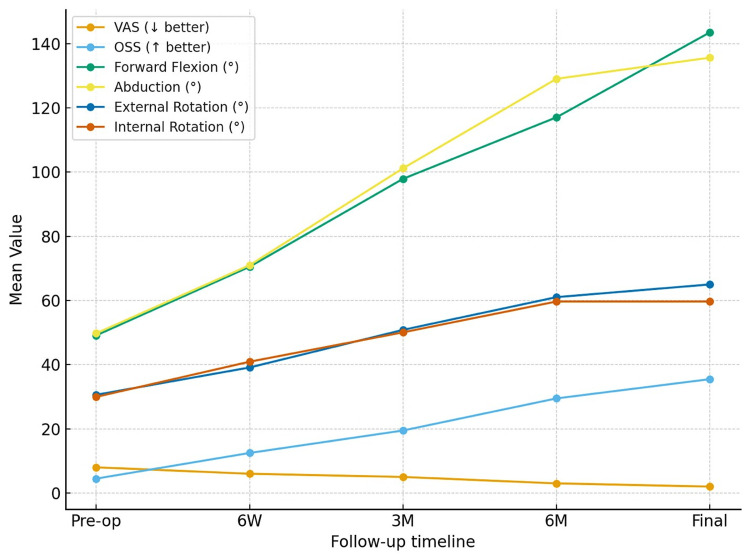
Sequential improvement in pain scores, functional outcome, and range of shoulder motion following JUST-UNIC fixation, demonstrating progressive recovery from the preoperative period through six-week, three-month, six-month, and final follow-up assessments.

Progressive improvement was noted throughout follow-up in pain scores, functional recovery, and shoulder motion. VAS scores reduced from 8.00 ± 0.82 preoperatively to 2.00 ± 0.61 at final follow-up, while OSS increased from 4.48 ± 1.21 to 35.48 ± 1.18. Forward flexion improved from 49.12 ± 5.83° to 141.65 ± 11.99°, abduction from 49.76 ± 4.74° to 136.41 ± 12.64°, external rotation from 30.60 ± 5.86° to 65.47 ± 5.73°, and internal rotation from 29.96 ± 4.59° to 61.76 ± 6.31° (Table 2). These sequential improvements are illustrated graphically in Figure 3.

**Table 2:** Longitudinal clinical outcomes (mean ± SD).

Outcome	Preoperative	Six weeks	Three months	Six months	Final follow-up
VAS (0-10)	8.00 ± 0.82	6.00 ± 0.81	5.00 ± 0.74	3.00 ± 0.72	2.00 ± 0.61
Oxford Shoulder Score (OSS, 0-48)	4.48 ± 1.21	12.48 ± 1.13	19.48 ± 1.26	29.48 ± 1.17	35.48 ± 1.18
Forward flexion (°)	49.12 ± 5.83	71.12 ± 6.83	97.12 ± 9.95	114.88 ± 11.67	141.65 ± 11.99
Abduction (°)	49.76 ± 4.74	71.76 ± 6.18	98.76 ± 12.69	126.47 ± 11.94	136.41 ± 12.64
External rotation (°)	30.60 ± 5.86	38.60 ± 7.02	49.60 ± 6.64	58.71 ± 5.74	65.47 ± 5.73
Internal rotation (°)	29.96 ± 4.59	39.96 ± 6.33	48.96 ± 7.18	61.76 ± 6.31	61.76 ± 6.31

**Figure 4:**
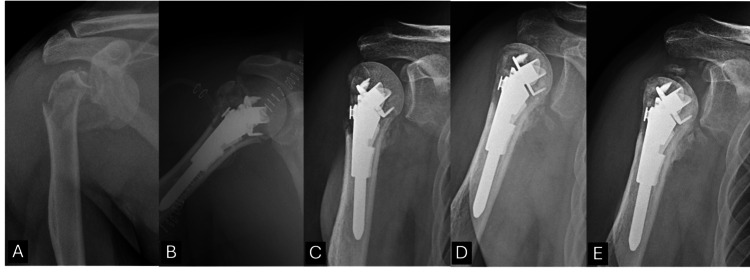
AVN after JUST UNIC fixation. (A) Preoperative X-ray. (B) Immediate postoperative X-ray. (C) Follow-up X-ray at six months showing united fracture and partially resorbed greater tuberosity. (D) Follow-up X-ray at 12 months showing sclerotic changes in the humeral head. (E) Illustrative postoperative radiograph at 25 months showing progression to avascular necrosis (AVN) with collapsed humeral head. Images beyond 12 months are shown for illustrative purposes only and were not included in the formal outcome analysis.

Postoperative complications occurred in four patients. Comparative outcome analysis between the complication group and uneventful-recovery group showed no major differences in final VAS, OSS, or ROM parameters (Table 3). A representative case of AVN progression following fixation is presented in Figure 4.

**Table 3:** Final outcome comparison based on presence of postoperative complications (n = 25).

Outcome (final)	No complication (n = 21)	Complication present (n = 4)	p-value
VAS final (0-10)	1.85 ± 0.91	2.50 ± 0.57	0.210
OSS final (0-48)	35.52 ± 1.07	34.25 ± 1.50	0.119
Flexion final (°)	142.05 ± 10.96	134.25 ± 9.11	0.197
Abduction final (°)	137.19 ± 11.58	129.75 ± 11.03	0.201
External rotation final (°)	65.19 ± 5.64	62.50 ± 7.42	0.441
Internal rotation final (°)	60.76 ± 6.41	57.75 ± 7.32	0.387

**Figure 5:**
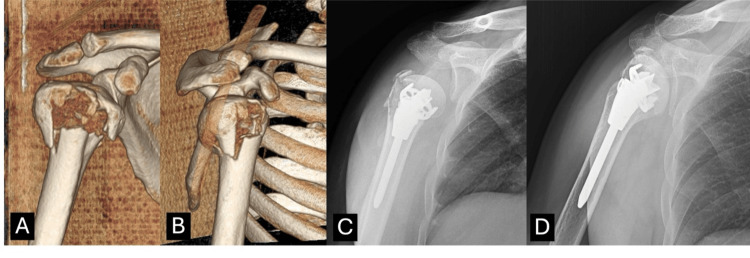
GT resorption after JUST UNIC fixation. (A, B) Preoperative CT images showing the fracture pattern. (C) Postoperative X-ray showing intact GT. (D) Final follow-up X-ray at 12 months showing united fracture with resorbed greater tuberosity. GT - greater tuberosity

Subgroup assessment based on GT resorption demonstrated similar patterns of pain improvement (Table 4) and OSS recovery (Table 5) between resorption and non-resorption groups. A radiographic sequence of tuberosity resorption with maintained union is shown in Figure 5.

**Table 4:** VAS progression across follow-up in GT resorption vs. no-resorption. GT - greater tuberosity

VAS score (0-10)	GT resorption yes (n = 15)	GT resorption no (n = 10)	p-value
Preoperative	7.9 ± 0.8	8.1 ± 0.9	0.60
Six weeks	5.9 ± 0.9	6.0 ± 0.7	0.74
Three months	4.7 ± 0.8	4.9 ± 0.6	0.53
Six months	2.7 ± 0.7	2.9 ± 0.6	0.47
Final follow-up	1.9 ± 0.6	2.0 ± 0.5	0.62

**Table 5:** OSS progression across follow-up in GT resorption vs. no-resorption. GT - greater tuberosity; OSS - Oxford Shoulder Score

Oxford Shoulder Score (0-48)	GT resorption yes (n = 15)	GT resorption no (n = 10)	p-value
Preoperative	4.5 ± 1.1	4.9 ± 1.2	0.41
Six weeks	11.5 ± 1.8	12.2 ± 1.7	0.28
Three months	18.5 ± 1.9	19.4 ± 1.6	0.22
Six months	28.8 ± 2.1	29.6 ± 1.8	0.31
Final follow-up	34.7 ± 1.9	35.3 ± 1.6	0.33

A total of four postoperative complications (16%) were observed in this series. One patient (4%) developed AVN with radiographic collapse by 12 months; however, at final follow-up, the patient remained functionally compensated, and no revision procedure had been performed during the study period. Varus malalignment (<125°) occurred in one patient (4%), but it did not result in any functional limitation. Another patient (4%) demonstrated partial staple prominence, which remained asymptomatic and did not require intervention. A superficial wound hematoma was noted in one case (4%) and was successfully managed with surgical washout, healing uneventfully thereafter. Importantly, no cases of implant breakage, loss of fixation, deep infection, or neurovascular complications were observed.

## Discussion

This study aimed to evaluate the clinical, functional, and radiological outcomes of patients with comminuted proximal humeral fractures involving the anatomical neck treated using the JUST UNIC™ device. At the end of follow-up, all fractures achieved radiological union within six months, indicating reliable osteosynthesis. Functional recovery was substantial, with mean VAS scores improving from 8.0 preoperatively to 2.0 at final follow-up and OSS increasing from 4.48 to 35.48, reflecting significant pain reduction and restoration of function. Range of motion showed consistent improvement across all planes, confirming progressive rehabilitation. The complication rate remained low, with only one case of AVN and three minor postoperative events, none of which significantly impacted final outcomes. Partial GT resorption was noted in several cases but did not correlate with poorer functional results. The relatively high rate of GT resorption observed in this cohort is likely multifactorial. Possible contributors include altered stress distribution beneath the metaphyseal staple with relative stress shielding and the degree of initial fracture comminution. Importantly, despite radiographic evidence of resorption, functional outcomes were preserved, suggesting maintained construct stability. These findings suggest that JUST UNIC fixation provides stable biomechanical support, facilitates early motion, and yields excellent union and functional outcomes with a low incidence of AVN, underscoring its clinical effectiveness for complex proximal humeral fractures involving the anatomical neck.

Comparison with existing literature

The findings of this study are in strong agreement with the expanding evidence base supporting bilboquet-derived fixation concepts, including JUST UNIC™ devices, for anatomically demanding PHFs involving the humeral neck. Previous clinical series have consistently reported reliable union, favourable functional outcomes, and reduced complication profiles compared to traditional fixation systems. In our cohort, radiological union was achieved in all patients within six months, accompanied by marked improvement in pain and function (VAS 8.0 → 2.0; OSS 4.48 → 35.48). These outcomes are comparable to those summarized by Samargandi et al. [14], who reported mean Constant scores of 69.7 and AVN incidence of 19.7% across 235 bilboquet-treated fractures, signifying reproducibility across clinical settings. Likewise, Bismuth et al. [15] demonstrated significantly higher functional scores and lower complication rates in the bilboquet group compared to locking plates, further establishing the reliability of this fixation philosophy. Our union rate (100%) and low AVN rate (4%) fall at the favourable end of this spectrum, reinforcing the biomechanical validity of the JUST UNIC construct.

The mechanical advantage of JUST UNIC fixation can be attributed to its integral design characteristics, namely, the Morse taper head-stem articulation, controlled valgus alignment, and secure metaphyseal purchase, which collectively distribute load efficiently across the fracture and limit varus collapse. The staple interface facilitates fixation of small articular segments at the anatomical neck, a region where plate-only constructs often struggle due to limited bone stock and vascular risk. These elements mirror foundational bilboquet principles described in earlier literature, emphasizing valgus maintenance, minimal periosteal disruption, and preservation of humeral head perfusion as determinants of successful outcomes.

Comparative evidence further highlights the limitations of locking plates in anatomical-neck fractures, where rates of varus failure, screw cut-out, and AVN remain notable, reported as high as 55% in certain anatomical-neck fracture cohorts. Porschke et al. [16] observed higher revision rates and complications following ORIF in elderly patients when compared to arthroplasty or bilboquet-derived implants, despite similar functional scoring. Long-term PHILOS outcomes have similarly shown persistent AVN rates of approximately 12%, as described by Birsel et al. [17], suggesting that anatomical reduction alone does not always ensure vascular protection. These findings emphasize the need for fixation models that provide both mechanical and biological protection criteria inherently met by JUST UNIC through metaphyseal stability, valgus contouring, and vascular preservation.

The JUST UNIC™ construct preserves the principal advantage of the bilboquet concept, achieving stable fixation while minimizing disruption to the humeral head vascularity. Its valgus-supporting configuration, aligning with the principles highlighted by Park and Seok [18], maintains head alignment under load and mitigates the risk of varus collapse, thereby enabling safer and earlier mobilization. Technical elements include FiberWire-reinforced tuberosity capture, secure head-shaft coupling through a Morse taper junction, and a low-profile metaphyseal staple that maximizes surface purchase-function synergistically to preserve rotational stability and permit progressive rehabilitation. When interpreted alongside current clinical performance and supporting literature, these biomechanical attributes position JUST UNIC™ as a reliable fixation option for fractures involving the anatomical neck, offering structural durability with comparatively lower biological compromise.

Clinical implications

The results of this study demonstrate that the JUST UNIC device is a viable and effective alternative for managing comminuted proximal humeral fractures involving the anatomical neck, particularly in middle-aged patients where arthroplasty is not ideal. Its unique bilboquet-inspired design offers the dual advantage of stable metaphyseal fixation and preservation of humeral head vascularity, enabling early rehabilitation and excellent shoulder function. The high union rate (100%) observed in this cohort, along with significant improvement in VAS and OSS, and minimal complications, suggests that this implant can reliably restore anatomy and biomechanics while minimizing risks associated with conventional locking plates, such as varus collapse, screw cut-out, and AVN. Furthermore, the ability to adjust the valgus angle intraoperatively allows surgeons to maintain proper head-shaft alignment, addressing one of the key failure points in standard fixation methods. The findings support integrating JUST UNIC fixation into the treatment algorithm for anatomically challenging PHFs, especially those with small head fragments or disrupted medial hinges, where traditional plates provide suboptimal stability.

Strengths and limitations

A key strength of this study lies in its focus on a highly complex and underreported fracture pattern, anatomical neck-involved comminuted proximal humeral fractures, using a novel fixation device with a clear biomechanical rationale. The prospective documentation of serial clinical and radiological outcomes, including objective functional scoring (VAS and OSS) and range of motion tracking, provides robust evidence of the implant’s efficacy. Moreover, the uniform surgical technique performed by experienced surgeons and consistent postoperative rehabilitation protocol minimize variability in outcomes. However, certain limitations must be acknowledged. The retrospective nature of the study introduces potential selection bias, and the absence of a control group prevents direct statistical comparison with other fixation methods. The final follow-up duration of 12 months is sufficient to evaluate fracture union and early functional recovery; however, it may underestimate the true incidence of late-onset AVN, which has been reported to manifest up to two to three years after injury. These factors should be considered when interpreting the results, and longer-term, comparative studies are needed to fully define implant-specific outcomes.

## Conclusions

This study establishes that the JUST UNIC fixation system provides excellent clinical, functional, and radiological outcomes in the management of comminuted proximal humeral fractures involving the anatomical neck. The device’s design effectively addresses the key challenges associated with these fractures-fragment instability, risk of varus collapse, and compromised vascularity-by combining intramedullary support with stable articular fixation through the bilboquet mechanism. The observed 100% union rate, substantial improvements in shoulder function, and low complication rate underscore its ability to achieve durable fixation while preserving humeral head viability. In this cohort, GT resorption was not observed to correlate with inferior clinical or functional outcomes. However, given the limited sample size, larger studies are required to confirm whether GT resorption has a meaningful impact on long-term shoulder function.

These findings apply specifically to patients aged ≤60 years with adequate bone quality and should not be extrapolated to elderly osteoporotic populations, in whom reduced metaphyseal purchase may compromise staple-based fixation. In this single-centre retrospective series, JUST UNIC fixation appeared safe and reliable, with excellent union and functional outcomes in anatomically challenging PHFs. These promising results merit confirmation in larger comparative studies with longer follow-up.
